# Sleep, anxiety and fatigue in family members of patients admitted to the intensive care unit: a questionnaire study

**DOI:** 10.1186/cc12736

**Published:** 2013-05-24

**Authors:** Alex Day, Samer Haj-Bakri, Stephanie Lubchansky, Sangeeta Mehta

**Affiliations:** 1 Department of Medicine and Interdepartmental Division of Critical Care, Mount Sinai Hospital, 600 University Avenue, Toronto, ON M5G 1X5, Canada; 2 University of Limerick, Medicine, Castletroy, Limerick, Ireland; 3 University of Ottawa, Medicine, 75 Laurier Avenue East, Ottawa, ON K1N 6N5, Canada; 4 University of Toronto, 100 St. George Street, Toronto, ON M5S 3G3, Canada

**Keywords:** sleep, fatigue, anxiety, family members, intensive care unit, questionnaire study

## Abstract

**Introduction:**

Family members of critically ill patients often experience increased incidence of physical and mental health issues. One of the first ways family members suffer is by losing sleep. The purpose of this study is to understand sleep quality, levels of fatigue and anxiety, and factors contributing to poor sleep in adult family members of critically ill patients.

**Methods:**

A questionnaire was designed to evaluate sleep, fatigue and anxiety during the intensive care unit (ICU) admission. We incorporated three validated instruments: General Sleep Disturbance Scale (GSDS), Beck Anxiety Index (BAI) and Lee Fatigue Scale (NRS-F). Adult family members of patients in ICU for more than 24 hours were approached for questionnaire completion. Patient demographics were recorded.

**Results:**

The study population consisted of 94 respondents, (49.1 ± 12.9 years, 52.7% male); 43.6% were children and 21.3% were spouses of ICU patients. Sleep quality was rated as poor/very poor by 43.5% of respondents, and good/very good by 15.2%. The most common factors contributing to poor sleep were anxiety (43.6%), tension (28.7%) and fear (24.5%). Respondents' most common suggestions to improve sleep were more information regarding the patient's health (24.5%) and relaxation techniques (21.3%). Mean GSDS score was 38.2 ± 19.3, with 58.1% of respondents experiencing moderate to severe sleep disturbance. Mean BAI was 12.3 ± 10.2, with 20.7% of respondents experiencing moderate to severe anxiety. Mean NRS-F was 3.8 ± 2.5, with 57.6% of respondents experiencing moderate to high fatigue. Family members who spent one or more nights in the hospital had significantly higher GSDS, BAI and NRS-F scores. The patient's Acute Physiology and Chronic Health Evaluation (APACHE) II score at survey completion correlated significantly with family members' GSDS, BAI and NRS-F.

**Conclusion:**

The majority of family members of ICU patients experience moderate to severe sleep disturbance and fatigue, and mild anxiety.

## Introduction

Critical illness and admission to an intensive care unit (ICU) is a traumatic experience that can lead to significant upheaval in the lives of both the patients and those closest to them. Since health professionals are traditionally trained to focus on the needs of the patient, the needs of the family may be overlooked [1,2]. The unfortunate reality is that family members of critically ill patients often experience increased incidence of physical and mental health issues, and are unlikely to prioritize their own needs [3]^.^

Following patient admission to the ICU, one of the first ways family members suffer is by losing sleep [4,5]. Poor sleep has been identified as an important factor in the physical and mental health of caregivers, and can result in changes in stress response, irritability, depression, diminished attentiveness, decreased immune function and compromised decision-making ability [6-11]. Although many studies have examined the impact of ICU admission on the sleeping patterns of patients, few have evaluated the sleep of family members while their loved ones are patients in the ICU [12-14]. It is important that health care professionals are aware of the ICU admission-related psychological morbidity in family members of ICU patients, as their ability to make decisions on behalf of the patient or provide care after discharge can be impaired [15,16]. The objective of this study was to better understand, using a self-administered questionnaire, the quality of family members' sleep, their levels of fatigue and anxiety, and factors contributing to poor sleep quality.

## Materials and methods

The study was conducted between July 2010 and September 2011 in the adult medical-surgical intensive care unit of Mount Sinai Hospital, a 16-bed ICU affiliated with the University of Toronto; and was approved by the institutional Research Ethics Board.

### Questionnaire (appendix 1)

Sleep, fatigue and anxiety were identified as the areas of chief concern in our study population, which were family members and close friends of ICU patients. We designed a questionnaire that asked respondents about their demographics, their relationship to the patient, previous sleep behaviors and diagnosed sleep disorders. For the time during the ICU admission we asked respondents about where they slept (home, hospital, hotel and so on), their perceived sleep quality and quantity, factors contributing to poor sleep and potential relieving factors for poor sleep. We also asked about the patient's location prior to ICU admission (for example, home, hospital ward and so on). Patient data included APACHE II scores on ICU admission and on the day of questionnaire completion, and intubation status.

A validated self-report tool was used to assess each area of interest: the General Sleep Disturbance Scale (GSDS), the Beck Anxiety Inventory (BAI), and Lee's Numerical Rating System for Fatigue (NRS-F) [17-19]. The GSDS includes 21 items that examine the frequency of sleep difficulties experienced during the past week, poor quality of sleep, daytime sleepiness and use of substances to help induce sleep. Each item is rated on an 8-point scale from 0 (never) to 7 (every day). Total scores of 0 to 29, 30 to 59, and ≥60 indicate mild, moderate and severe sleep disturbance, respectively [17]. The BAI examines 21 common symptoms of anxiety and the respondent is asked to rate the severity of each symptom from 0 (not at all) to 3 (severe) [18]. Total scores of 0 to 21, 22 to 35 and ≥36 indicate mild, moderate and severe anxiety, respectively. The NRS-F is an 18-item instrument which assesses the level of fatigue and energy in both normal and patient populations. Each of the 18 items measures the presence of a characteristic of fatigue or energy on a scale from 0 (not at all) to 10 (extremely) and the total score represents the overall average [20]. Total scores of 0 to 3.2, 3.3 to 6.5, and ≥6.6 indicate low, moderate and high fatigue, respectively. For questionnaire completion we asked respondents to restrict their responses to the duration of time since their loved one had been admitted to the ICU (please see Additional file 1 for the questionnaire).

### Questionnaire administration

All available family members and friends of patients who met the following criteria were approached for survey completion: any individual ≥18 years who was a relative or close friend of an ICU patient, including parent, spouse, offspring, sibling or member of the patient's household; and whose relative/friend had been in the ICU for at least 24 hours. A research associate approached those who met criteria in the ICU waiting room or at the bedside. The study design was explained to each participant and the research associate obtained verbal agreement for questionnaire completion. Once the relative/friend completed the anonymous questionnaire, it was returned to the research associate or deposited in a mailbox. For each ICU patient, up to three relatives/friends were approached for survey completion.

### Statistical analysis

Data are presented as means and standard deviations or percentages. Kolmogorov-Smirnov normality tests and Pearson's correlations were used to examine the different measures (that is, APACHE II on admission and at the time of survey completion, GSDS, BAI and NRS-F). One-way ANOVAs with Tukey's b *post-hoc *tests were used to compare respondents in different groups. Analyses were carried out using IBM SPSS Statistics Version 20 (Chicago, IL, USA). A *P*-value of ≤0.05 was considered statistically significant. We tested the validity of each of our tools for our sample population using a Cronbach Alpha test for internal consistency. The NRS-F and BAI each received excellent internal consistency scores (Cronbach Alphas of 0.93 and 0.92, respectively), and the GSDS had good internal consistency (Cronbach Alpha of 0.80).

## Results

The questionnaire was completed by 100 individuals, 6 questionnaires were excluded due to incompleteness, resulting in 94 surveys representing 72 patients. The mean number of days between the patient's admission to the ICU and questionnaire completion was 4.6 ± 7.6. Respondent demographics and patient APACHE II scores are presented in Table 1. Of the 94 respondents, 43.6% were children, and 21.3% were spouses of ICU patients. Of the patients, 71.3% were intubated and mechanically ventilated; APACHE II was lower on the day of questionnaire completion than on the day of ICU admission.

**Table 1:** Respondent and patient demographics

Respondent demographics (N = 94)	
Age, years	49.1 ± 12.9
Male, N (%)^a^	49 (52.7)
Relationship to patient, N (%) Child Spouse Parent Friend Other Sibling	41 (43.6) 20 (21.3) 11 (11.7) 9 (9.6) 8 (8.5) 5 (5.3)
Diagnosed sleep disorder, N (%)	9 (9.6)
Patient demographics (N = 72)	
Mechanically ventilated, N (%)	55 (71.3)
APACHE II on admission	24.5 ± 9.7
APACHE II on survey date	18.6 ± 8.0

This table shows respondent and patient demographic data. Data are presented as mean ± SD or N (%). APACHE II, Acute Physiology and Chronic Health Evaluation II score. ^a^One respondent did not report their gender.

**Figure 1:**
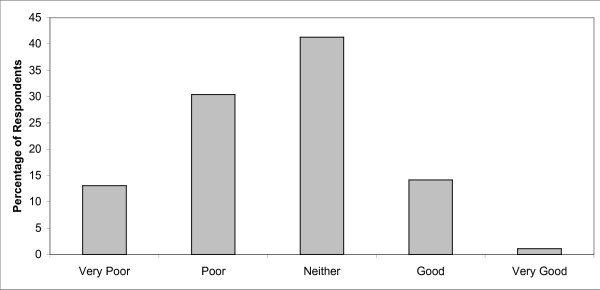
**Self-reported quality of family members' sleep during their loved one's ICU admission**.

**Figure 2:**
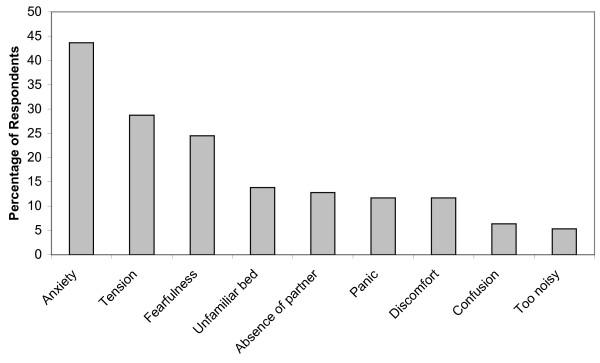
**Self-reported factors affecting a family member's sleep while their loved one was in the ICU**.

**Figure 3:**
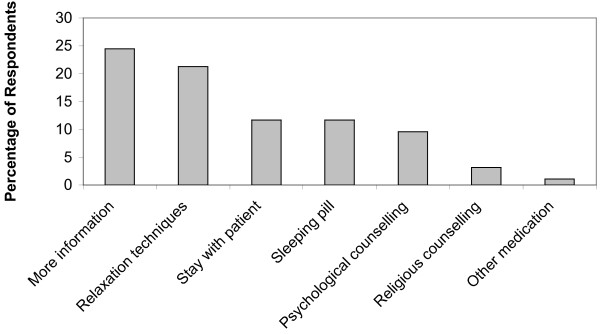
**Self-reported remedies suggested by family members to improve their sleep quality**.

Figure 1 shows respondents' perceived sleep quality while their loved ones were patients in the ICU. Of the 94 respondents, 66.0% reported having difficulty sleeping, 43.5% described sleep quality as poor or very poor, and only 15.1% described it as good or very good. Prior to their family member's ICU admission, 93.6% of respondents reported having a normal sleep/wake cycle; however, only 41.5% of subjects had a normal sleep/wake cycle during their loved ones ICU stay. The three most common factors reported as being responsible for poor sleep were anxiety (43.6%), tension (28.7%) and fearfulness (24.5%) (Figure 2). Family members were asked what interventions they believed would help improve their sleep; 78 (83%) responded, and the most commonly suggested remedies were more information regarding their loved one's health (24.5%) and relaxation techniques (21.3%) (Figure 3).

Table 2 shows mean GSDS, BAI and NRS-F scores, and the severity of impairment of each symptom. The mean GSDS was 38.2 ± 19.3, with 58.1% of respondents reporting moderate to severe sleep disturbance. The mean BAI score was 12.3 ± 10.2; and 20.7% of respondents had moderate to severe anxiety. The mean NRS-F score was 3.8 ± 2.5, with 57.6% of respondents experiencing moderate to high levels of fatigue.

**Table 2:** The GSDS, BAI, and NRS-F scores and severity of impairment

Scale	Score N = 93^a^	Mild/Low impairment N (%)	Moderate impairment N (%)	Severe/High impairment N (%)
GSDS	38.2 ± 19.3	39 (41.9)	39 (41.9)	15 (16.1)
BAI	12.3 ± 10.2	73 (79.4)	17 (18.5)	2 (2.2)
NRS-F	3.8 ± 2.5	39 (42.4)	42 (45.7)	11 (12.0)

This table shows mean scores for the GSDS, BAI and NRS-F and the percentage of respondents who scored mild/low, moderate or severe/high impairment for each scale. Scores are presented as mean ± SD. **^a^**N for GSDS is 93, N for BAI and NRS-F is 92. BAI, Beck Anxiety Index; GSDS, General Sleep Disturbance Scale; NRS-F, Lee Fatigue Scale

Table 3 shows correlations between the patient's APACHE II scores on admission and at the time of questionnaire completion, and the GSDS, BAI and NRS-F scores. The APACHE II score on admission was not correlated with the GSDS, BAI or NRS-F; however, the APACHE II score at the time of questionnaire completion correlated significantly with the GSDS, BAI and NRS-F. Further, the GSDS, BAI and NRS-F all correlated significantly with one another. There was no correlation between a patient being intubated and mechanically ventilated and respondents' GSDS, BAI or NRS-F scores (*P *= 0.725, 0.368, 0.834, respectively).

**Table 3:** Correlations between patients' APACHE II scores and the family members' GSDS, NRS-F and BAI

		Admission APACHE II	Survey APACHE II	GSDS	BAI	NRSF Fatigue
Admission APACHE II	Pearson Correlation	1	0.636	0.106	0.026	0.059
	*P*-value	--	<0.001	0.154	0.402	0.286
Survey APACHE II	Pearson Correlation	0.636	1	0.253	0.197	0.202
	*P*-value	<0.001	--	0.007	0.028	0.025
GSDS	Pearson Correlation	0.106	0.253	1	0.513	0.554
	*P*-value	0.154	0.007	--	<0.001	<0.001
BAI	Pearson Correlation	0.026	0.197	0.513	1	0.587
	*P*-value	0.402	0.028	<0.001	--	<0.001
NRS-F	Pearson Correlation	0.059	0.202	0.554	0.587	1
	*P*-value	0.286	0.025	<0.001	<0.001	--

This table shows correlations between the patients' APACHE II scores at the time of admission and at the time of questionnaire completion and the family members' GSDS, NRS-F, and BAI scores. All *P-*values are one-tailed.

Admission APACHE II is the APACHE II score at the time of patient's admission to the ICU. Survey APACHE II is the APACHE II score on the day that the family member completed the questionnaire. BAI, Beck Anxiety Index; GSDS, General Sleep Disturbance Scale; NRS-F, Lee Fatigue Scale

Respondents who did not live with the patient (N = 43) had significantly higher GSDS (*P *= 0.050) and BAI (*P *= 0.041) scores, but not NRS-F (*P *= 0.772), compared with respondents who were living with the patient prior to ICU admission (N = 51). All relationship groups (child, spouse, parent and so on) were equally affected regarding sleep, fatigue and anxiety (GSDS, BAI, NRS-F) by admission of a loved one; however, children of a patient were significantly more fatigued when compared to friends (*P *= 0.022).

The most common locations that the family members slept while their loved one was admitted to the ICU were at home (57.4% of nights), in the waiting room (22.3% of nights), and at a hotel (18.1% of nights). The two most frequently reported reasons for sleeping in the hospital were that their home was too far from the hospital (29.8%), and they were too anxious to leave the hospital (19.1%). Twenty-seven percent of respondents reported sleeping in the ICU waiting room overnight, and those family members who spent at least one night sleeping in the hospital (N = 25) had significantly higher GSDS (*P *= 0.015), BAI (*P *= 0.003), and NRS-F (*P *= 0.009) scores than family members who never slept overnight in the hospital. The impact of patient location prior to the ICU admission on family member's sleep quality, fatigue and anxiety was also examined. Family members of patients admitted from home had significantly lower GSDS scores (*P *= 0.013) compared with patients transferred to the ICU from another location within the hospital or from another hospital.

## Discussion

The objectives of this study were to assess how adult relatives of ICU patients were sleeping, their levels of fatigue and anxiety, and the factors contributing to poor sleep quality. The results of our questionnaire study indicate that family members of critically ill patients experience poor sleep, moderate fatigue and mild anxiety. More than 65% of respondents reported having difficulty sleeping during their family member's ICU admission, and 43.5% rated their sleep quality as poor or very poor.

Our findings support previous studies showing that family members of ICU patients report poor sleep quality and quantity [4,20,21]. In the study by Van Horn and Tesh, 70% of 50 adult family members of ICU patients reported worse sleep quality compared with sleep prior to the ICU admission, and 80% slept less than their usual amount [4]. In the study by Halm *et al*., 36% of 52 family members of ICU patients reported worse quality sleep and 44.8% reported reduced sleep quantity, compared with the time prior to ICU admission [21].

### Reasons for poor sleep

There are many factors responsible for poor sleep in family members of critically ill patients. In our study, family members reported anxiety, tension and fearfulness as the three most common reasons for poor sleep. Emotional stimulants, such as anxiety, fear and tension, have been shown to negatively affect a person's ability to fall asleep [22]. The high prevalence of anxiety in family members' of ICU patients has been well documented, with studies reporting rates of anxiety ranging from 35% to 73% [23-26]. In our study, the mean BAI score indicated mild anxiety in the majority of relatives, and 43.6% of family members reported anxiety as a cause of sleep disturbance.

### How to improve sleep: more information

We found that sleep disturbance, anxiety and fatigue all correlated with one another, thus it is possible that treatment of one factor may lead to improvement of the others. For example, providing consistent information to the patient's family regarding their loved one may reduce anxiety and improve sleep. Respondents supported this notion as "more information about my family member's health" was the most common choice selected for improving their sleep. The need for more information and a greater frequency of updates has also been cited by other studies as a possible solution for reducing anxiety and promoting family members' sleep [4,15,27,28]. Pochard *et al*. concluded that the lack of regular meetings with a physician or nurse was significantly associated with an increased risk of anxiety in family members [24].

### How to improve sleep: relaxation techniques

The second most common suggestion to improve the sleep of family members was relaxation techniques, including meditation and visual imagery, which have been shown to be effective in previous studies [5,29]. Although anxiety, fear and tension are to be expected when a family member is critically ill, practicing relaxation techniques may reduce the impact that these feelings have on overall emotional well-being, as well as sleep [5]. Pamphlets can be placed in ICU waiting rooms that warn about the consequences of sleep deprivation and recommend specific relaxation techniques. Moreover, ICU health care professionals should encourage family members to practice self-care, including relaxation techniques and other sleep-promoting activities.

### The impact of sleep location

The results of our study indicate that the location where family members sleep is a major factor in their sleep disturbance, anxiety and fatigue. Those respondents who spent one or more nights sleeping in the hospital had more sleep disturbance (GSDS), anxiety (BAI) and fatigue (NRS-F) than those who spent no nights sleeping in the hospital. Spending nights in the ICU waiting room is far from a rarity, as 27% of respondents reported sleeping there overnight. The regularity with which family members sleep overnight in the ICU was further supported by Van Horn and Tesh, who reported that 52% of family members spent at least one night sleeping in the waiting room [4]. The need to remain close to the patient is a major reason family members choose to sleep in waiting rooms. In our study, 19.1% of respondents stayed overnight in the waiting room because they were too anxious to leave. The correlation between the frequency of sleeping in the hospital and sleep disturbance, anxiety and fatigue, may suggest that hospital accommodations available for family members are inadequate for quality sleep.

### APACHE II score correlation

Not surprisingly, the patients' APACHE II scores on the day of survey completion was found to correlate significantly with the GSDS, BAI and NRS-F of family members. However, the APACHE II scores at the time of ICU admission had no significant correlation with the GSDS, BAI and NRS-F. From this we can infer that the patient's current severity of illness is a critical factor in sleep, fatigue and anxiety levels in our respondent population.

### Harmful consequences of sleep deprivation

The adverse effect of sleep deprivation on decision-making abilities is a serious issue for family members who must make critical decisions regarding their loved one's care. Several studies have outlined the harmful effects of short and long term sleep loss, which can include poor concentration, and poor quality of life [5]. As well, high levels of negative emotions, such as anxiety, can interfere with information recall and rational decision-making [15]. Furthermore, the harmful effects of sleep deprivation can impact the ability of family members to provide for the patient's health care needs after discharge. With the current trend towards earlier discharge of patients, the burden of home care often falls on the patients' family members [30]. It is clear from our study, as well as previous studies, that sleeplessness, anxiety and other health problems are prevalent in family members of ICU patients [16]. These health problems will only be exacerbated once they are designated as the full-time caregiver for their loved one.

### Strengths and limitations

Our study is one of very few to examine the sleep quality of family members of critically ill patients [4,21]. The strengths of our study include the large number of family-member respondents compared to other surveys, and the use of validated scales to assess sleep disturbance, anxiety and fatigue.

There were several limitations of our study. Self-report questionnaires have inherent limitations, including respondent recall bias and selection bias, since those family members who were the most affected by their loved one's critical illness may have elected not to complete the questionnaire. Another limitation was that we did not evaluate family members' sleep, anxiety and fatigue following discharge of the patient from the ICU to determine if these symptoms improved as the critical illness resolved.

## Conclusions

In this questionnaire study, the majority of family members of ICU patients experienced moderate to severe sleep disturbance and fatigue, along with mild anxiety. Given the harmful consequences of sleep deprivation and the importance of the mental and physical health of family members in their roles as substitute decision-makers and later as caregivers, it is clear that the sleep quality and general psychological health of family members is a significant issue. Some of these symptoms may be improved with better communication between the ICU team and family members, and the provision of more information regarding the patient. As well, ICUs should strive to provide better sleeping accommodations and support for family members regarding self-care and relaxation techniques.

## Key messages

• The majority of family members of ICU patients experience moderate to severe sleep disturbance and fatigue, along with mild anxiety.

• There are many factors responsible for poor sleep in family members of critically ill patients. In our study, family members reported anxiety, tension, fearfulness and location of sleep as the most common reasons for poor sleep.

• Some of these symptoms may be improved with better communication between the ICU team and family members, the provision of more information regarding the patient and updating ICU waiting rooms with cots and blankets.

• The adverse effects of sleep deprivation on family members may interfere with their decision making abilities while their loved one is in the ICU, as well as interfere with their care-taking ability after patient discharge.

## Abbreviations

APACHE II: Acute Physiology and Chronic Health Evaluation II; BAI: Beck Anxiety Index; GSDS: General Sleep Disturbance Scale; NRS-F: Numerical Rating Scale for Fatigue: also known as the Lee Fatigue Scale

## Competing interests

The authors declare that they have no competing interests.

## Authors' contributions

AD carried out data processing, data analysis and wrote the manuscript. SH conceived of the study, designed the questionnaire, collected data and performed the statistical analysis. SL conceived of the study, designed the questionnaire and collected data. SM coordinated the design of the study and helped to draft the manuscript. All authors have read and approved the final manuscript.
